# Feature replacement methods enable reliable home video analysis for machine learning detection of autism

**DOI:** 10.1038/s41598-020-76874-w

**Published:** 2020-12-04

**Authors:** Emilie Leblanc, Peter Washington, Maya Varma, Kaitlyn Dunlap, Yordan Penev, Aaron Kline, Dennis P. Wall

**Affiliations:** 1 Department of Pediatrics, Stanford University, Palo Alto, CA 94305 USA; 2 Department of Bioengineering, Stanford University, Palo Alto, CA 94305 USA; 3 Department of Computer Science, Stanford University, Palo Alto, CA 94305 USA; 4 Department of Biomedical Data Science, Stanford University, Palo Alto, CA 94305 USA; 5 Department of Psychiatry and Behavioral Sciences (by courtesy), Stanford University, Palo Alto, CA 94305 USA

**Keywords:** Machine learning, Autism spectrum disorders, Paediatric research

## Abstract

Autism Spectrum Disorder is a neuropsychiatric condition affecting 53 million children worldwide and for which early diagnosis is critical to the outcome of behavior therapies. Machine learning applied to features manually extracted from readily accessible videos (e.g., from smartphones) has the potential to scale this diagnostic process. However, nearly unavoidable variability in video quality can lead to missing features that degrade algorithm performance. To manage this uncertainty, we evaluated the impact of missing values and feature imputation methods on two previously published autism detection classifiers, trained on standard-of-care instrument scoresheets and tested on ratings of 140 children videos from YouTube. We compare the baseline method of listwise deletion to classic univariate and multivariate techniques. We also introduce a feature replacement method that, based on a score, selects a feature from an expanded dataset to fill-in the missing value. The replacement feature selected can be identical for all records (general) or automatically adjusted to the record considered (dynamic). Our results show that general and dynamic feature replacement methods achieve a higher performance than classic univariate and multivariate methods, supporting the hypothesis that algorithmic management can maintain the fidelity of video-based diagnostics in the face of missing values and variable video quality.

## Introduction

Autism Spectrum Disorder (ASD) is a complex neuropsychiatric condition affecting an estimated 53 million children under 5 years old worldwide and one million children in the US ten years of age or younger^1–4^. Autism’s prevalence in the U.S. rose from 1 in 125 to 1 in 40 children within the last 5 years alone^5,6^. Although early ASD diagnosis is critical to the outcome of behavior therapies before sensitive periods of development have passed, families, particularly in rural and underserved communities, still face waiting times exceeding 12 months^59,60^, repeated visits to professionals and out of state trips to establish a final diagnosis^61^.

Trained on electronic medical records archived from standard-of-care diagnostic assessments, artificial intelligence algorithms have shown the ability to classify ASD and the potential to reduce waiting times for diagnosis, optimize caregivers’ work load, and reach previously underserved populations through digital health tools. Using the Social Responsiveness Scale (SRS)^7^, Duda et al. applied feature selection^8,9^ to develop an ASD vs Attention Deficit Hyperactivity Disorder classification algorithm, achieving high performance (Area Under the Receiver Operating Characteristic curve (AUC-ROC) = 96.5%). Washington et al. built upon this work to develop an ASD vs neurotypical (NT) neural network classifier, achieving an AUC-ROC of over 92% for SRS-derived diagnoses^10^. Leveraging Autism Diagnostic Observation Schedule (ADOS)^11^ scoresheet records, Küpper et al. developed ASD vs NT algorithms, yielding an AUC-ROC of 90% on patients under 21 years old^12^. Levy et al.^13^ similarly found reduced sets of features highly predictive for ASD in ADOS Module 2 and Module 3 datasets. Autism Diagnostic Interview-Revised (ADI-R)^14^ scoresheets have also been used for developing ASD classifiers. Duda et al.^15^ used ADOS scoresheets to create an algorithm exhibiting >97% sensitivity and >77% specificity in comparison to ADOS-based diagnosis. Wall et al.^16^ applied feature selection and found that 7 of the 93 items contained in the ADI-R were sufficient to classify autism, achieving 99.9% statistical accuracy with a 7-question classifier using the ADI-R score sheets of 966 individuals. This feature selection work suggests that the number of features can be reduced from 30 questions per module for the standard instrument ADOS (4 modules, 116 questions total) and 93 questions for the standard instrument ADI-R, to 9 or 7 features depending on the model used. This potentially translates to decreased time to diagnosis as well as mutually beneficial opportunities to use alternative modes of feature capture, even by non-experts evaluating video from mobile devices. Finally, the use of these features in models opens opportunities to move the diagnosis from a binary outcome to a more continuous, quantitative measure that can be tracked over time.

Subsequent experiments focused on independent validation using novel independent datasets confirm that the models retain high performance, supporting their generalizability and limiting the potential of overfitting issues. Wall et al.’s 7-feature algorithm achieved 84.8% Unweighted Average Recall (UAR; the mean of the sensitivity and specificity) when tested on a prospective sample of 222 children^17^. This same model was also validated by Bone et al.^18^ on a dataset with reasonable balance of autism and non-autism developmental delay cases (BID)  of 680 child participants, producing a UAR of 80% in comparison to the clinical standard outcome. Bone et al. also tested an 8-feature alternating decision tree model from Wall et al.^31^ in a different slice of their BID containing 1,033 children and found 94% UAR against the standard clinical diagnosis. Tariq et al.^19^ validated several logistic regression models on an independent dataset of 1,089 individuals with autism and 66 individuals with no autism diagnosis, achieving 94.1% UAR with 9 features. These experiments support the hypothesis that small numbers of features can be used by machine learning models to produce an accurate classification of autism. 

However, moving models to practice for more efficient and accessible autism diagnoses requires methods for rapid and reliable feature measurements. Guided by frameworks such as the one proposed by Stark et al.^20^, such algorithms are now being integrated as quantitative classification tools into real world settings and embedded into mobile and telemedicine solutions. Leveraging YouTube videos, Fusaro et al. showed that features used in today′s gold standard assesssment tools, such as the ADOS,  can be measured with high accuracy from viewing short videos of children^21^. Abbas et al. trained two algorithms to identify autism from mobile inputs: one based on structured parent-reported questionnaires and the other on tagging key behaviors from short, semi-structured home videos of children^22,23^. Their results outperformed baseline clinician screeners^24^. Tariq et al.^19^ evaluated the performance of 8 ADOS- and ADI-R-trained machine learning algorithms—including the osmicki et al.^25^ and Wall et al.^16^ algorithms that we leverage in this paper—on ratings by blinded non-experts of 5-min home videos of children with and without ASD, all achieving over 94.5% sensitivity. To render these "human-in-the-loop" algorithms scalable, Washington et al. leveraged crowdworkers^58^ to rapidly obtain the necessary video ratings for minimal feature sets to run models with highest autism classification accuracy^26^ and integrated privacy-protection measures to the process^27^.  This work has shown the high potential for using mobile video and machine learning models for more scalable, affordable, and repeatable diagnosis; yet, important questions remain on how variability in video content impacts feature measurement.

The algorithms mentioned above rely on a fixed set of features, such as the ability to maintain eye contact or the presence of repetitive speech, and their prediction performance decreases when one or more of these features is not measurable ^28^. During an observational assessment of a child at risk for autism, features can be missing for numerous reasons: children may be unable to express certain behaviors (e.g., because of their age), videos used to capture the child’s interactions may only display a subset of the features needed (e.g., video length too short or quality too low) and some raters may not understand or may feel uncertain when answering specific questions. To translate machine learning algorithms into everyday healthcare, researchers must develop a robust missing feature strategy. Much like a clinician, an algorithm should adapt to the child’s capacities and cannot experience a significant drop in performance if certain features are not available. For example, if “Repetitive Speech” is a feature of the algorithm and it cannot be evaluated on non-verbal children, alternative features with the same predictive power should be used instead and safeguards should be implemented to avoid misclassifications. Feature imputation and NULL-value treatment methods have been analyzed for healthcare- and psychiatry-related classifiers. Abidin et al. compared the performance of three machine learning classifiers (k-nearest neighbors, decision tree, and Bayesian networks) for data imputation accuracy^29^. Aisha et al. analyzed the effect of 9 different missing value treatments on the accuracy of four Bayesian network classifiers used to predict death in acute chronic Hepatitis patients^30^. However, most ASD-related papers are centered around overall algorithm performance, only briefly mentioning the type of feature imputation technique (for instance, Küpper et al. mention leveraging 5 nearest neighbors^12^).

In this paper, we use and evaluate two previously published ASD classification models: logistic regression with 9 features (LR9)^25^ and alternating decision tree with 7 features (ADTree7)^16^. We evaluate various methods of treating missing values on the performance of these algorithms, training them on standard-of-care instrument scoresheets (ADOS Module 2 scoresheets for LR9 and ADI-R version 2003 scoresheets for ADTree7) and testing using non-expert ratings of 140 children YouTube videos. We compare (1) standard univariate and multivariate techniques for feature imputation with (2) general feature replacement strategies and (3) dynamic feature replacement methods which adapt to each specific record. Our work highlights the potential of imputation techniques for video-based ASD classification algorithms and the broader potential for use of feature replacement strategies in remote, mobile diagnostics.

## Materials and methods

All methods described below were carried out in accordance with global, federal, state, and university guidelines and regulations for research and reviewed and approved by the Stanford University Institutional Review Board (IRB) prior to taking place.

### Models

This work relies on a set of previous experiments, building towards the creation of a novel and scalable video-based assessment of ASD. The first set of experiments used electronic medical record data from standard-of-care measurements made during the clinical diagnosis of autism (or the absence of such diagnosis)^16,19,31^. This work focused on feature selection and dimensionality reduction to train 8 models and test their accuracy against the clinical decision for the child at risk for a developmental delay, including autism^19^. Kosmicki et al.^25^, Levy et al.^13^ and Washington et al.^10^ illustrated how feature selection methods can reduce the number of standard-of-care instrument questions needed from 30 questions per module for ADOS (4 modules, 116 questions total) and 93 for ADI-R, to 9 or 7 behavioral features (depending on the model used), all while preserving high model performance. These experiments documented that the features needed for autism diagnosis can be significantly fewer than what is used in today’s standard-of-care. The next experiment showed that the feature vectors needed for the models can be objectively measured in home videos of the child at risk for autism. To do so, our team has developed a secure video rating portal in which raters can view short home-video clips of children and submit their answers. In total, non-clinician raters score 30 behavioral features per video to ensure coverage of all 8 machine learning model features analyzed by Tariq et al.^19^. Finally, Tariq et al.^19^, Duda et al.^9^ and Washington et al.^26^ have shown that prediction accuracy was preserved with scores collected through this system, when based on home-video clips with variable manifestations of autism and other developmental delays^8^. Tariq et al.^32^ have also explored the adaptability of these models to distinct cultures and countries. Together this body of prior work (a.) found the optimal features and models, and (b.) demonstrated the ability to run the models on home videos quickly, suggesting that autism diagnosis may be possible through video scoring. The present takes the critical next step to address the robustness of the video diagnostic process to variability in home video quality and content.

Variability in video length and quality remains a great challenge for the scalability of this ASD screening system, particularly as it increases the risk of missing values—raters being unable to assess some behavioral features. Our goal is to manage and limit missing values in these scores, all while decreasing rating time (i.e. the number of questions) and adapting to the specific content of each video.

This study focuses on and evaluates two published machine learning ASD diagnosis algorithms: logistic regression with 9 features (LR9)^25^ and alternating decision tree with 7 features (ADTree7)^16^. LR9 features are "Expressive Language", "Eye Contact", "Joint Attention/Pointing", "Stereotyped Speech", "Spontaneous Gestures", "Indicates Pleasure to Others", "Social Overtures", "Complex Mannerisms" and "Stereotyped Interests/Actions". ADTree7 features are "Expressive Language", "Understands Language", "Eye Contact", "Developmental Delay", "Social Participation", "Pretend Play" and "Indicates Pleasure to Others" (see Supplementary Table S4 for summarizing table). Both models were validated by subsequent independent experiments achieving 98.9% sensitivity and 89.4% specificity for LR9, and at worst 89.9% sensitivity and 79.7% specificity for ADTree7^17,18^. LR9 was tested on independent data from 1,089 individuals with ASD and 66 individuals with no ASD diagnosis and ADTree7 was validated in a clinical trial of 222 participants and in a reasonably balanced independent dataset consisting of 680 individuals (462 with ASD). These models are also structurally quite different. LR9 and ADTree7 rely on two distinct families of machine learning models (Logistic Regression being a linear classifier and Decision Trees a non-linear classifier), they are trained on two different instruments (ADOS Module 2 and ADI-R 2003) and only share 3 common features. Because of these differences, the feature imputation and feature replacement methods’ performances are susceptible to vary widely between LR9 and ADTree7. Comparing their performances on these two models thus offers a better assessment of the methods’ quality.

### Datasets

#### Training dataset

Following approval by the Stanford University IRB, our training dataset^33^ was assembled and analyzed. This dataset groups de-identified ADOS and ADI-R electronic health records previously collected by multiple sources: Autism Genetic Resource Exchange^34^, Autism Consortium, National Database for Autism Research^35^, Simons Simplex Collection^36^, Simons Variation in Individuals Project^37^. Under an IRB approved data transfer agreement between Stanford University and Cognoa Inc., we also included a previously collected dataset of de-identified ADI-R responses from Cognoa Inc. As the datasets described above were secondary data sources, informed consent was waived by our IRB.

ADI-R consists of 93 items: 2 free-response items, 14 age of onset items, and 77 ordinal scale items whose responses range from 0 (typical behavior) to 4 (severely atypical). ADOS is administered as four different modules, with each module being appropriate for a different age range and child ability. Responses range from 0 (typical behavior) to 3 (severely atypical). As defined in the initial development of these models^19^, we only use ADOS Module 2 for LR9 training and ADI-R 2003 for ADTree7 training. This groups a total of 16,200 instrument ratings, of which 4,343 are ADOS Module 2 score sheets (Supplementary Tables S1a and S2a) and 11,857 ADI-R 2003 score sheets (Supplementary Tables S1b and S2b). The balance of males to females in the dataset matches the increased prevalence of ASD in males compared to females^38^.

#### Testing dataset

With the same methods for video data collection and feature tagging as described in Tariq et al.^19^, we collected 140 publicly available YouTube videos of children, 70 ASD and 70 NT balanced for age and gender (Supplementary Tables S3a and S3b). As we collected publicly available data, collection of informed consent was waived by Stanford University IRB. Videos were selected from YouTube using YouTube metatags to confirm the age and diagnosis of the child in the video. If a video did not include a metatag for the age of the child in the video, the age was assigned following full agreement among the estimates made by 3 clinical practitioners in pediatrics. Videos were selected based on whether the video (1) was between 1 and 5 minutes in length, (2) showed the face and hands of the child, (3) showed clear opportunities for or direct social engagement, and (4) involved opportunities for the use of an object such as a utensil, crayon, or toy.

As in Tariq et al.^19^, ratings were performed by either students (high school, undergraduate, or graduate-level) or working professionals with no formal training or certification for detection or diagnosis of autism. The rating questionnaire consisted of 30 behavioral features (e.g., eye contact, social smile), used in previously published machine learning models and shown to be highly predictive of ASD^19^ (see Supplementary File Table S4 for the detailed list of features). All raters received minimal training with a trained clinical coordinator prior to performing feature ratings and were blind to the diagnosis of the child in the video. The testing dataset used here included 3 distinct ratings chosen at random from this pool of untrained raters for each of the 140 videos (i.e. 3 distinct 30-feature vectors per video). No optimization was conducted based on the raters’ previous performance nor based on rater types. We use this dataset of 3 ratings of the 30 features for each of the 140 5-min YouTube videos as our test set.

### Missing values core concepts

Little and Rubin^39^ introduce 3 categories of missing data: (1) missing completely at random (MCAR), (2) missing at random (MAR) and (3) non-ignorable. In MCAR, the probability of missingness is the same for all records. If a variable is MCAR, then ignoring records with missing data should not introduce bias. Ignoring records with missing data (i.e., listwise deletion) is our baseline method for addressing missing values. For MAR cases, the probability of missingness varies for each record but remains predictable from other variables in the database. For example, if we assume males are less likely to fill-in the mental health part of a medical survey, this will induce higher missing values but would be unrelated to the status of their mental health. Therefore, we can fill-in missing values appropriately (i.e. predict mental health responses) by considering the variables affecting the probability of missingness (i.e. gender since it affects the probability of mental health responses’ missingness). However, it is difficult to ensure MAR as there may be unobserved variables also influencing the process. We consider our features to be MAR when we predict missing values based on the other features of the algorithm. Finally, in the non-ignorable case, data missingness is non-random, depending on information that has not been recorded in the database. By definition, this makes it extremely difficult to identify and predict. To address this, we expand the dataset we use to predict the missing values to other variables, even if they were not included in the original model’s features, through general and dynamic feature replacement techniques. Although this dataset expansion helps reduce risk, it does not entirely rule out the existence of another latent variable. To these three missing data classifications, we can add a fourth: (4) missingness that depends on the missing value itself. For instance, in a survey, the probability of having a missing value in a salary question will most likely depend on the salary amount itself. This introduces a high risk for bias. As our datasets are composed of ratings of videos done by individuals with no particular link to the child in the video, we do not expect them to engage in such self-censoring behaviors.

### Methodology

**Figure 1:**
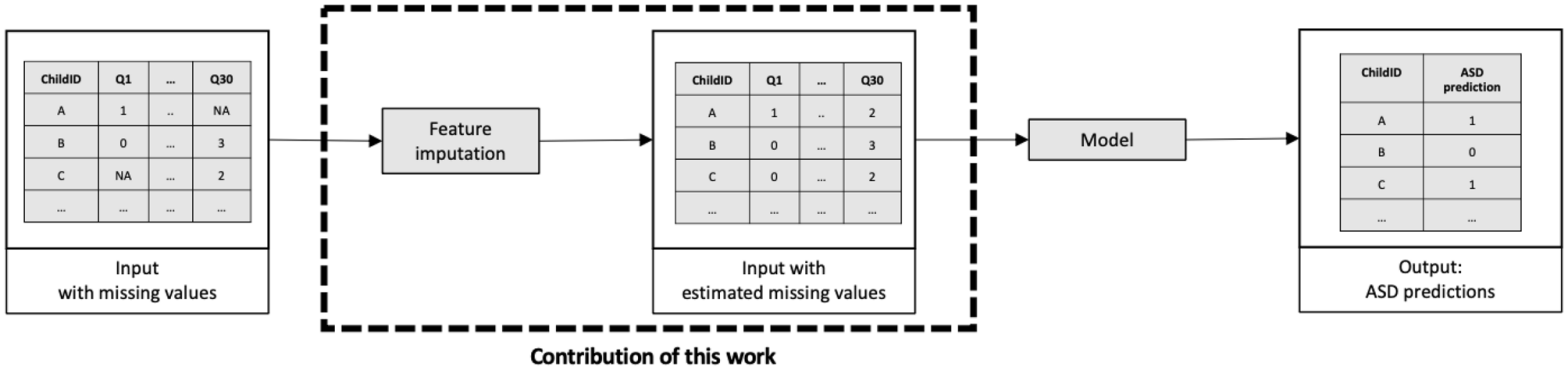
Pipeline description and contribution.

We describe a pipeline of employing a feature imputation method and feeding the resulting features into a diagnostic classification model (Fig. 1). Our study focuses on finding the best feature imputation method for ASD classification (see Supplementary File “Additional Information—Mathematical Formulation”).

#### Evaluation

**Figure 2:**
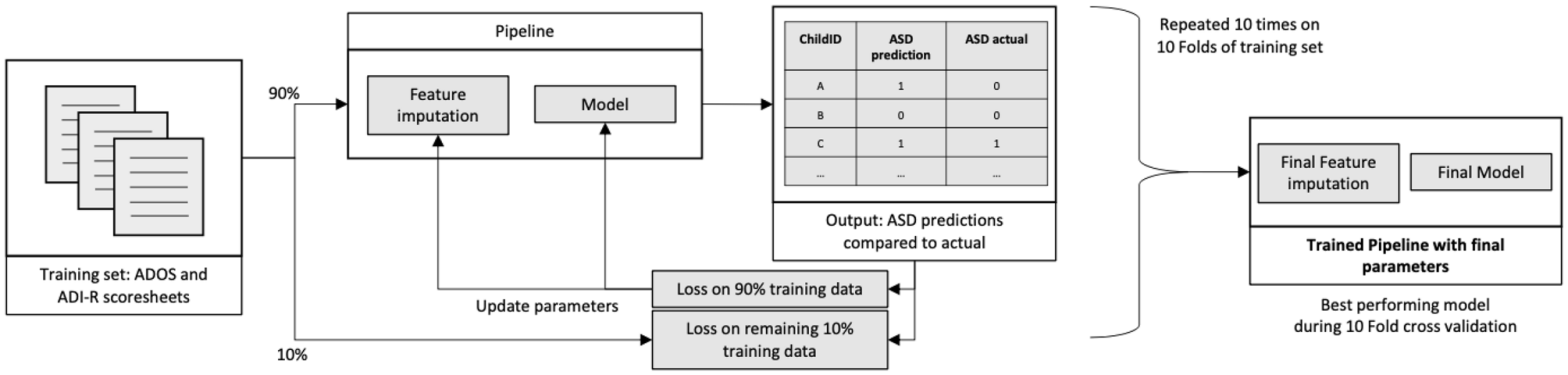
Pipeline training process on instrument scoresheets.

**Figure 3:**
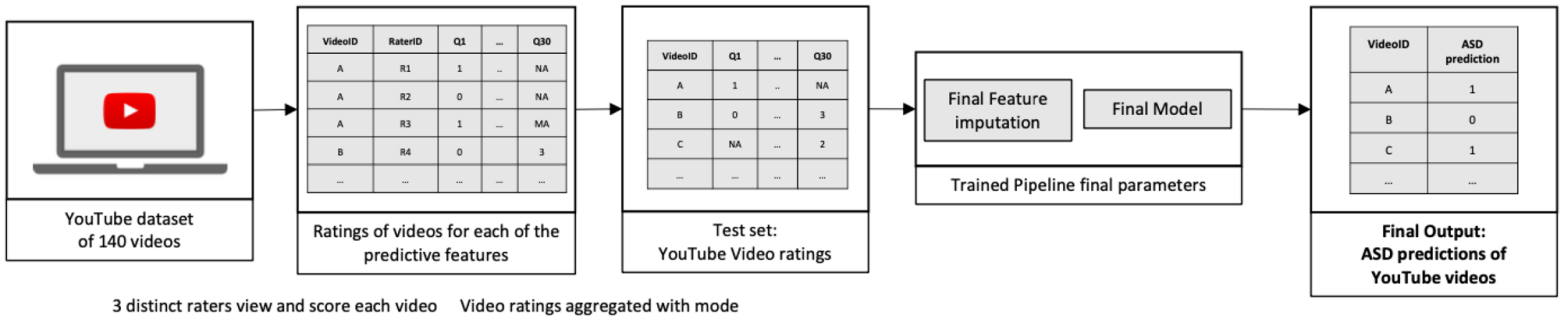
Pipeline testing process on YouTube video ratings.

We compare LR9 and ADTree7 model performances with each feature imputation method. For every model and every feature imputation method, all items of the pipeline are trained, each time adapting the feature imputation method and tuning model hyperparameters using a 10-fold GridSearch cross validation optimizing for UAR (Fig. 2). The trained pipeline is then tested on the YouTube dataset (Fig. 3), with ratings for each video aggregated with mode (i.e., most frequent value) using the *scikit-learn*^40^ library in *Python 3*. This process is repeated 5 times to account for the variability in the generated folds and to be able to report on average and standard deviation performance.

### Feature imputation methods compared

#### Baseline: listwise deletion

Listwise deletion simply omits records containing missing values. Although it is often the default method in analyses, listwise deletion involves losing information and may introduce a bias if missing values are not missing completely at random (MCAR). It is considered as the baseline algorithm in this paper and we compare the performance of each feature replacement method to this baseline. In our case, a record will be dropped from the training set if at least one of the model’s features is NULL and we will not be able to attribute a prediction to the test record if all of the 3 raters have answered NULL in at least one same feature.

#### Classic feature imputation techniques: univariate

Univariate feature imputation methods replace missing values in a feature with a constant obtained by aggregating non-missing values of that same feature. We compare 3 different statistics: **mean**, **median** and **most frequent value** (i.e. mode).

#### Classic feature imputation techniques: multivariate

Multivariate feature imputation techniques rely on all the model features to fill missing values. In our case, this corresponds to all 9 features for LR9 and all 7 features for ADTree7. In an iterative round-robin fashion, we predict the missing values in feature $j^{*}$ based on all other *j* features such that $j \ne j^{*}$ and $j \in [1,n]$ and *n* the number of features in the model. We compare 3 commonly used techniques: **Gaussian mixture with expectation-maximization**^41^, **ridge regression**^42^, and **decision trees**^43^ (see Supplementary File “Additional Information—Classic feature imputation techniques: Multivariate”).

#### General feature replacement methods

**Figure 4:**
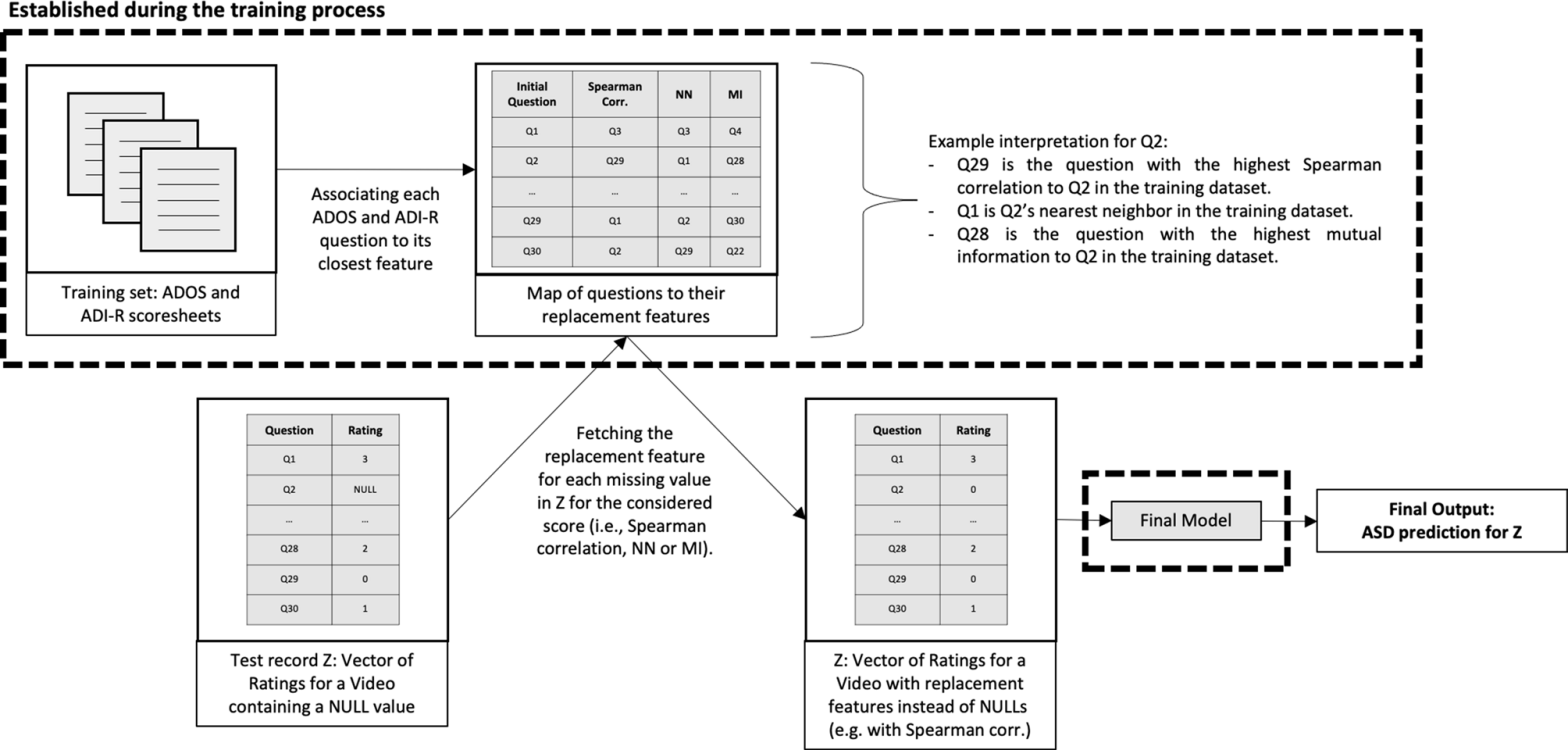
General feature replacement process illustration.

We expand the feature space by considering all features available in ADOS Module 2 or ADI-R 2003, not limiting ourselves to the 9 or 7 features of the previously published models. Instead of creating a model that would rely on a combination of these features, we simply attempt to replace the missing value with another “close” feature’s value as described in Fig. 4. Although this enhances the feature space mathematically, in practice, this method allows us to replace (and not add) a question a rater may be unable to answer with the next best question. This maintains the initial number of questions asked to raters and simultaneously adapts the questionnaire to the video content if a behavioral feature cannot be assessed.

Therefore, we map all *n* (= 9 or 7) features of the model to their closest feature in the larger set of $n^{*}$ features available according to a score *s* (e.g., mutual information (MI)) (see Supplementary File “Additional Information—Mathematical Formulation—General feature replacement methods”). We compare three different scores to select the replacement feature: **correlation**-based, **nearest neighbor** (NN), and **mutual information**-based. Correlation-based selects the feature with the highest Spearman correlation with the feature we wish to fill. Because the input features (i.e. questions answered by raters) are ranked on an ordinal scale, we chose Spearman correlation as it does not assume a linear distribution and measures how well the relationship between two variables can be described as a monotonic function^44^. Similarly, the nearest neighbor method selects the closest nearest neighbor feature as its replacement using Euclidean distance. Finally, MI-based feature replacement selects the feature having the highest MI with the missing feature.

#### Dynamic feature replacement

**Figure 5:**
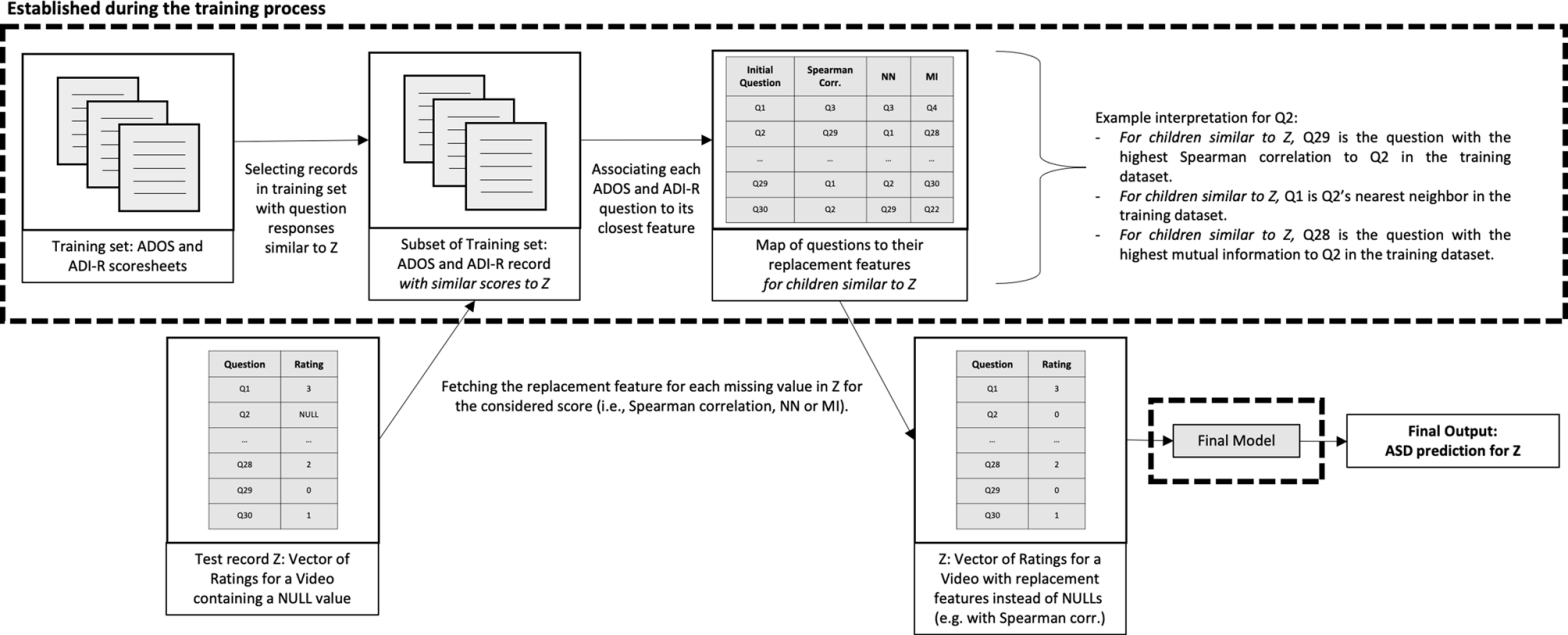
Dynamic feature replacement process illustration.

In this final method, we realize that the best replacement feature (selected via correlation, NN or MI) may vary depending on the child being rated and the contents of the video. To take this into consideration, for each new test record, we dynamically apply the feature replacement methods described above on a subset of the training set corresponding to records having similar ratings as the test set (described in Fig. 5; see also Supplementary File “Additional Information—Mathematical Formulation—Dynamic feature replacement”).

## Results

### Dataset analysis

We notice a drastic difference in the percentage of missing values in each of the model’s training sets: the LR9 model features in the ADOS Module 2 dataset contain on average 2.3% of missing values (Supplementary Figure S1a) while that average increases to 17.8% for ADTree7 (Supplementary Figure S1b). This difference is reduced in testing as the testing dataset is identical for both models. However, the average percentage of missing values per feature for LR9 is still slightly lower at 5.1% (Supplementary Figure S2a) compared to 8.9% for ADTree7 (Supplementary Figure S2b), as the models have different input features. Missing values tend to be concentrated in one or two features: for LR9, “Joint Attention Pointing” and “Spontaneous Gestures” are missing for 13.8% and 14.8% of ratings respectively (Supplementary Figure S2a) and, for ADTree7, “Pretend Play” is missing for over 40.7% of our ratings (Supplementary Figure S2b). We then analyzed the origin of the missing values in our YouTube testing dataset with Welch t-tests and Bonferroni correction. We compared the overall average number of missing values and the number of missing values per feature, between ASD and NT children, between age groups, and between genders. Although no significant difference was noted between the populations in the overall number of missing values, some individual features had an associated *p* value $< 0.05$. The following features have significantly more missing values in the ASD population than in the NT population at level of significance $\alpha$ = 0.05: "Echolalia" (*p* value = 0.0235, not significant after Bonferroni correction), "Speech Patterns" (*p* value = 6.8623e−08), "Communicative Engagement" (*p* value = 1.4198e−05), "Understands Language" (*p* value = 2.3683e−05) and "Stereotyped Speech" (*p* value = 0.0008). No feature was significant at $\alpha$ = 0.05 when comparing female and male participants and, when comparing age groups, only "Imitates Actions" had significantly more missing values in the 1 to 3 year old age group (*p* value = 0.0455), although this does not stand after Bonferroni multiple hypothesis correction. This analysis shows that missing values tend to occur more often for ASD children than NT children in specific features ("Speech Patterns", "Communicative Engagement", "Understands Language", "Calls Attention to Objects", "Stereotyped Speech", "Stereotyped Interests and Actions") and that the diagnosis of the child is an unobservable latent variable influencing their missingness. Throughout this paper and as a proxy, we impute missing values with the help of features that are themselves predictors of ASD vs NT diagnosis.

### Baseline listwise deletion

In the YouTube dataset, 135 ratings have at least one missing value in the LR9 features and 205 ratings have at least one missing value in the ADTree7 features. As we apply listwise deletion as a baseline, these ratings were dropped, thus reducing the number of ratings per video to 1 or 2 (since we started with 3 ratings per video). 5 videos for LR9 and 21 videos for ADTree7 could not be rated at all as at least one feature was missing in all 3 of the ratings of these videos. The model achieves an average UAR of 82.38% (0.0117 standard deviation) for LR9 and 84.47% (0.0265 standard deviation) as seen in Table 1. However, ADTree7 has a high variability in results, generating higher standard deviations, particularly increasing the Area Under the Precision-Recall curve (AUC PR). We use a Welch t-test for all results to evaluate the significance of the performance increase or decrease compared to baseline. A $^{*}$ symbol indicates significance at level $\alpha = 0.05$ and a $^{*B}$ symbol indicates the significance at level $\alpha = 0.05$ stands after Bonferroni correction.

**Table 1:** Baseline performance of LR9 and ADTree7 on 420 ratings of 140 YouTube videos (average performance and standard deviation). With listwise deletion, a rating is dropped if it contains at least one NULL value in the model's features (this is the case for 135 ratings for LR9). We are unable to rate a video if at least one model feature is missing in all 3 ratings of this video (this is the case for 5 videos for LR9).

Model	Sensitivity	Specificity	UAR	AUC-ROC	AUC PR	Ratings dropped
LR9	0.8939 (0.0152)	0.7536 (0.0102)	0.8238 (0.0118)	0.9109 (0.0021)	0.9195 (0.0018)	135 ratings and 5 videos entirely
ADTree7	0.8172 (0.0397)	0.8721 (0.0709)	0.8447 (0.0261)	0.9083 (0.0117)	0.8706 (0.0644)	205 ratings and 21 videos entirely

### Classic feature imputation techniques

#### Univariate

All univariate feature imputation methods yield significant improvements for LR9 for all metrics except sensitivity (Table 2a). Sensitivity only significantly improves for median and mode methods, although this does not stand after Bonferroni correction. Median achieves the best UAR of the univariate feature imputation methods with 88.29% (0.0096). However, univariate feature imputation methods do not perform as well for ADTree7, which contains more NULL values than LR9. The only significant improvements are achieved by mean and median in specificity and do not stand after Bonferroni correction (Table 2b). The top UAR of 83.29% (0.0760) is achieved with mean feature imputation but does not match the baseline model’s performance.

**Table 2:** Univariate feature imputation methods—performance of LR9 and ADTree7 on 420 ratings of 140 YouTube videos.

(a) Performance of LR9 (average performance and standard deviation).					
Method	Sensitivity	Specificity	UAR	AUC-ROC	AUC PR
Mean	0.9029 (0.0064)	0.8571$^{*B}$ (0.0226)	0.8800$^{*B}$ (0.0128)	0.9541$^{*B}$ (0.0012)	0.9658$^{*B}$ (0.0013)
Median	0.9143$^{*}$ (0.0000)	0.8514$^{*B}$ (0.0192)	0.8829$^{*B}$ (0.0096)	0.9577$^{*B}$ (0.0016)	0.9695$^{*B}$ (0.0010)
Mode	0.9171$^{*}$ (0.0064)	0.8400$^{*B}$ (0.0212)	0.8786$^{*B}$ (0.0113)	0.9569$^{*B}$ (0.0006)	0.9671$^{*B}$ (0.0002)

(b) Performance of ADTree7 (average performance and standard deviation).					
Method	Sensitivity	Specificity	UAR	AUC-ROC	AUC PR
Mean	0.6857 (0.1446)	0.9800$^{*}$ (0.0373)	0.8329 (0.0760)	0.9105 (0.0306)	0.8799 (0.1396)
Median	0.6057 (0.3307)	0.9714$^{*}$ (0.0143)	0.7886 (0.1694)	0.9150 (0.0142)	0.7810 (0.1247)
Mode	0.7000 (0.1325)	0.9457 (0.0445)	0.8229 (0.0859)	0.8823 (0.0591)	0.7646 (0.1548)

#### Multivariate

Multivariate imputation methods tend to perform as well as univariate imputation methods for LR9, except for feature imputation with Gaussian mixtures. Although ridge regression and decision trees both achieve more than 88.7% UAR, neither generate a jump in sensitivity significant enough for the Welch test (Table 3a). For ADTree7, decision trees seem to perform better than other multivariate methods, but it does not pass the significance test (Table 3b).

**Table 3:** Multivariate feature imputation methods—performance of LR9 and ADTree7 on 420 ratings of 140 YouTube videos.

(a) Performance of LR9 (average performance and standard deviation).					
Method	Sensitivity	Specificity	UAR	AUC-ROC	AUC PR
Gaussian mixture	0.9029 (0.0186)	0.8457 (0.1085)	0.8743 (0.0467)	0.9477$^{*B}$ (0.0074)	0.9604$^{*B}$ (0.0072)
Ridge regression	0.9114 (0.0064)	0.8657$^{*B}$ (0.0128)	0.8886$^{*B}$ (0.0064)	0.9549$^{*B}$ (0.0012)	0.9660$^{*B}$ (0.0012)
Decision trees	0.9057 (0.0128)	0.8686$^{*B}$ (0.0445)	0.8871$^{*B}$ (0.0185)	0.9576$^{*B}$ (0.0023)	0.9680$^{*B}$ (0.0015)

(b) Performance of ADTree7 (average performance and standard deviation).					
Method	Sensitivity	Specificity	UAR	AUC-ROC	AUC PR
Gaussian mixture	0.7429 (0.1313)	0.8514 (0.128)	0.7971 (0.0597)	0.8067 (0.0869)	0.7659 (0.1483)
Ridge regression	0.8229 (0.0594)	0.8886 (0.0356)	0.8557 (0.0321)	0.8937 (0.0508)	0.8724 (0.0782)
Decision trees	0.7571 (0.0562)	0.9829$^{*}$ (0.0235)	0.8700 (0.0222)	0.9289 (0.0353)	0.9214 (0.0806)

### General feature replacement techniques

General feature replacement methods use features that were rated but not included in the initial models as substitutes to model features if marked NULL. This methodology unlocks significant improvements that were not achieved with the classic feature imputation methods. For LR9, when using the nearest neighbor feature, we achieve a significant improvement for all of the metrics considered and only sensitivity does not pass the Bonferroni correction (Table 4a). For ADTree7, general feature replacement methods are the first to achieve significant performance with Bonferroni correction: the highest mutual information method yields 93.16% AUC-ROC and the nearest neighbor method reaches the 90.00% balanced accuracy mark (Table 4b). These methods also help analyze which questions are easier to rate. As seen in Supplementary Tables S5 and S9, “Quality of Social Overtures” is often replaced with “Amount of social overtures / maintenance of attention”, hinting that rating quantity may be easier than quality. We also note the presence of simpler concepts such as “Pointing” and “Showing”, which are preferred to concepts like “Complex mannerisms”, “Joint attention” and “Descriptive gestures”, which could help make the questions more accessible to non-expert raters.

**Table 4:** General feature replacement methods—performance of LR9 and ADTree7 on 420 ratings of 140 YouTube videos.

(a) Performance of LR9 (average performance and standard deviation).					
Method	Sensitivity	Specificity	UAR	AUC-ROC	AUC PR
Most correlated feature	0.9114 (0.0064)	0.8629$^{*B}$ (0.0239)	0.8871$^{*B}$ (0.0117)	0.9597$^{*B}$ (0.0012)	0.9708$^{*B}$ (0.0008)
Nearest-neighbor feature	0.9171$^{*}$ (0.0120)	0.8286$^{*B}$ (0.0286)	0.8729$^{*B}$ (0.0185)	0.9582$^{*B}$ (0.0028)	0.9696$^{*B}$ (0.0024)
Highest mutual information feature	0.9086 (0.0078)	0.8657$^{*B}$ (0.0192)	0.8871$^{*B}$ (0.0128)	0.9599$^{*B}$ (0.0010)	0.9706$^{*B}$ (0.0008)

(b) Performance of ADTree7 (average performance and standard deviation).					
Method	Sensitivity	Specificity	UAR	AUC-ROC	AUC PR
Most correlated feature	0.7771 (0.0217)	0.9886$^{*}$ (0.012)	0.8829$^{*}$ (0.0081)	0.9445$^{*}$ (0.0252)	0.9406 (0.0484)
Nearest-neighbor feature	0.8257 (0.031)	0.9743$^{*}$ (0.0293)	0.9000$^{*B}$ (0.0231)	0.9295 (0.0323)	0.9073 (0.0813)
Highest mutual information feature	0.7343$^{*}$ (0.0603)	0.9629$^{*}$ (0.0217)	0.8486 (0.0234)	0.9316$^{*B}$ (0.0054)	0.8150 (0.1454)

### Dynamic feature replacements

When applying our new dynamic feature replacement scheme on the YouTube dataset, we notice a significant improvement for all metrics but sensitivity for LR9 when compared to the base model, achieving a maximum of 89.57% UAR with dynamic mutual information (Table 5a). For ADTree7, we notice a significant increase of UAR compared to baseline, although passing the Bonferroni correction only with mutual information (Table 5b). When comparing general and dynamic feature replacement methods, we notice equivalent performances for LR9, overall equivalent UAR for ADTree7 and an increase in sensitivity for ADTree7. This new method therefore appears to maintain the high performances of the general feature replacement method on top of allowing for an automatic selection of the replacement feature. The increase in ADTree7 sensitivity may also indicate that including information on the individual record when choosing the best feature replacement reduces the imbalance in false positive vs false negatives.

**Table 5:** Dynamic feature replacement methods—performance of LR9 and ADTree7 on 420 ratings of 140 YouTube videos.

(a) Performance of LR9 (average performance and standard deviation).					
Method	Sensitivity	Specificity	UAR	AUC-ROC	AUC PR
Dynamic—most correlated feature	0.9171$^{*}$ (0.0839)	0.8286$^{*}$ (0.0156)	0.8729$^{*B}$ (0.0070)	0.9585$^{*B}$ (0.0018)	0.9690$^{*B}$ (0.0015)
Dynamic—nearest-neighbor feature	0.9171$^{*}$ (0.0057)	0.8543$^{*B}$ (0.0305)	0.8857$^{*B}$ (0.0143)	0.9596$^{*B}$ (0.0020)	0.9697$^{*B}$ (0.0016)
Dynamic—highest mutual information feature	0.9114 (0.0057)	0.8800$^{*B}$ (0.0280)	0.8957$^{*B}$ (0.0132)	0.9613$^{*B}$ (0.0033)	0.9711$^{*B}$ (0.0023)

(b) Performance of ADTree7 (average performance and standard deviation).					
Method	Sensitivity	Specificity	UAR	AUC-ROC	AUC PR
Dynamic—most correlated feature	0.8343 (0.0333)	0.9371 (0.0400)	0.8857$^{*}$ (0.0175)	0.9200 (0.0168)	0.9168 (0.0448)
Dynamic—nearest-neighbor feature	0.8429 (0.0239)	0.9343 (0.0400)	0.8886$^{*}$ (0.0154)	0.9164 (0.0117)	0.9325 (0.0079)
Dynamic—highest mutual information feature	0.8714$^{*}$ (0.0286)	0.9171 (0.0262)	0.8943$^{*B}$ (0.0177)	0.9120 (0.0356)	0.8871 (0.0658)

## Discussion

In anticipation of the widespread use of machine learning classifiers as detection tools for autism^45^, here we studied the impact of missing values on the performance of two previously published ASD classifiers, a logistic regression using 9 features (LR9) and an alternating decision tree model using 7 features (ADTree7), using a dataset of non-expert ratings of 140 YouTube child videos. We compared common univariate and multivariate feature imputation methods to general and dynamic feature replacement techniques. For LR9, general feature replacement methods achieve a similar performance as classic univariate and multivariate methods (general feature replacement methods achieve at best 88.71% UAR vs 88.86% for multivariate methods). However, when confronted with even more missing values, as is the case with ADTree7, general feature replacement methods achieve a higher average UAR than classic and multivariate approaches (general feature replacement methods achieve 90.00% UAR vs 87.00% for multivariate feature imputation). General feature replacement methods also help elucidate which questions are easier to rate and may point to ways to improve their formulation. Dynamic feature replacement methods allow a jump in average UAR for LR9 (achieving 89.57% when the replacement feature is dynamically selected via mutual information) and an improvement in sensitivity for ADTree7 (87.14% with replacement feature dynamically selected via mutual information). Overall, we see that using algorithmic-driven replacement questions in place of missing values and dynamically personalizing feature imputation methods to the YouTube video considered allows for an increase in UAR for both LR9 and ADTree7.

One main concern is the risk of overfitting in the general and dynamic feature replacement methods we introduced. Dynamic feature replacement in particular relies on a large training set containing enough diversity in children profiles to provide an accurate replacement feature to each test record considered. There is a risk for a drop in performance for rare rating vectors and for types of ratings significantly different from our training set (for instance if we consider raters or children from outside of the United States).

To limit the risk of overfitting, we used machine learning models constrained to have few features and assigned a lower bound to the regularization parameters during hyperparameter tuning while minimizing the model error. We also used 10-fold cross-validation, which enables sequential evaluation of the UAR with different partitions of the dataset. In addition, we were careful to use a gender and age balanced dataset (41.4% females, 58.6% males; 53.6% 1–3 y.o., 42.9% 4–6 y.o. children; Supplementary File Table 3) to avoid overfitting to one type of demographic. An important additional point is that prior experiments have shown that the LR9 and ADTree7 classifiers generalize to large independent datasets, achieving a sensitivity of 98.9% and 89.9% resp. and a specificity of 89.4% and 79.7% resp.^17–19^, therefore further minimizing their potential of overfitting.

Despite these safeguards, the overfitting risk introduced by the feature replacement methods remains. Methods such as shuffling labels, adding noise or randomly removing part of the training data can be helpful in measuring the impact of overfit. Other methods, such as bagging, help limit its impact. In particular, we welcome future work that tests these methods on independent datasets, as it is the best way to measure their tendency to overfit.

Future work is also needed to validate these findings using actual at-home videos instead of YouTube videos. Digital mobile^46–50^ and wearable^51–57^ therapeutics are increasingly collecting highly structured videos of children with ASD and matched neurotypical controls, such as unaffected siblings. This process is building the necessary video database for the validation of our methods. More work is also needed to validate our findings when using crowdsourced ratings. Indeed, realistic and representative videos of children in naturalistic settings can be fed into a crowdsourced pipeline leveraging the methods discussed in the present study to extract diagnostic features of children with autism, enabling for the remote, scalable, and speedy screening of ASD. Another important next step will be to compare the performance of feature replacement methods (both general and dynamic) to the rater’s intuition and “best guess” by prohibiting NULL values in ratings.

## Supplementary information

Supplementary Information.
