# Supplementary material for: Feature replacement methods enable reliable home video analysis for machine learning detection of autism
Source: Sci Rep. 2020 Dec 4;10:21245. doi: 10.1038/s41598-020-76874-w (PMC7719177; doi:10.1038/s41598-020-76874-w)
Supplement: Supplementary file 1 — Supplementary Information. [file 41598_2020_76874_MOESM1_ESM.pdf]

Feature replacement methods enable reliable home video analysis for machine learning detection of autism

Emilie Leblanc[1], Peter Washington[2], Maya Varma[3], Kaitlyn Dunlap[1,4], Yordan Penev[1,4], Aaron Kline[1,4], and Dennis P. Wall[1,4,*]

[1]Stanford University, Department of Pediatrics, Palo Alto, CA 94305, USA
[2]Stanford University, Department of Bioengineering, Palo Alto, CA 94305, USA
[3]Stanford University, Department of Computer Science, Palo Alto, CA 94305, USA
[4]Stanford University, Department of Biomedical Data Science, Palo Alto, CA 94305, USA
[*]dpwall@stanford.edu

SUPPLEMENTARY FILE

Additional Information

Classic feature imputation techniques: Multivariate

The first multivariate feature imputation method we selected is **ridge regression**. Ridge regression predicts the target value (here, the missing value) based on the other available features in the model's dataset. It includes a regularization parameter λ in the estimation procedure that reduces the risk of overfitting on the training set.

Gaussian mixtures assume that data points are generated from a mixture of K Gaussian distributions, i.e. clusters, each with unknown parameters (mean and standard deviation). Based on an estimation of these parameters, we are able to predict the missing values in our dataset. Our Gaussian mixture object implements the expectation-maximization (EM) algorithm for parameter estimation. The EM algorithm iterates between two modes: 1) the expectation step (E) fixes the Gaussian model parameters and computes the conditional probability of each data point according to these parameters and 2) the maximisation step (M) computes the Gaussian model parameters that maximize the probabilities found in E. Once clusters of data are learned, examples with missing entries can be identified as parts of a given cluster and completed with the most likely values given the non-missing entries for this cluster. We opted for this method since the significant difference in the number of missing values between ASD and NT in part "Results - Dataset analysis" implies that our data may live on separate clusters that unsupervised learning methods can discover.

Finally, **decision trees** are a non-parametric supervised learning method aiming to learn simple decision rules for prediction inferred from the data. Decision tree is another common predictive model used to impute missing values using approaches such as the *missForest*[1] technique that builds a random forest model for each variable.

Mathematical Formulation

With X, a dataset of n features and k records, let us note $x_{i,j}$ as the value of the j th feature for the i th record in X. We call Y the target binary vector containing the ASD diagnosis of all k records in X (0 corresponding to NT and 1 to ASD). In the current context, X corresponds to our ADOS or ADI-R training dataset and Y to the instrument-level ASD diagnosis. If X contains a missing value in position (i, j), we call $x_{i,j}^{\text{nomiss}}$ the actual value that should be in $x_{i,j}$ and $\hat{x}_{i,j}^{\text{nomiss}}$ our estimation of $x_{i,j}^{\text{nomiss}}$ through f_{imp}, the feature imputation method. We are looking for f_{imp} such that we minimize the error between X^{nomiss} (version of X containing all $x_{i,j}^{\text{nomiss}}$) and $\hat{X}^{\text{nomiss}}$ (our estimation of X^{nomiss} containing all $\hat{x}_{i,j}^{\text{nomiss}}$). However, since we do not have access to X^{nomiss} in practice (we do not know the "correct" value to fill NAs with) and since our main goal is the final accuracy of the model, we look for f_{imp} such that we maximize the unweighted average recall (UAR) between Y and $\hat{Y}$, our prediction of the ASD diagnosis from the model h (either LR9 and ADTree7) trained on $\hat{X}^{\text{nomiss}}$. We then test this process on Z, the video ratings containing missing values and for which we want to predict the ASD diagnosis. Similarly, if Z contains a missing value in feature j, we call z_j^{nomiss} the actual missing value and $\hat{z}_j^{\text{nomiss}}$ our estimation of z_j^{nomiss} through f_{imp}, feature

imputation method. To summarize, we look for f^*_{imp} such that:

$$f^*_{\text{imp}} = \underset{f_{\text{imp}}}{\arg\min}(\|X^{\text{nomiss}} - \hat{X}^{\text{nomiss}}\|) = \underset{f_{\text{imp}}}{\arg\min}(\|X^{\text{nomiss}} - f_{\text{imp}}(X)\|)$$

However, we do not have access to X^{nomiss}, so we use:

$$\begin{aligned} f^*_{\text{imp}} &= \underset{f_{\text{imp}}}{\arg\max}(UAR(Y,\hat{Y})) \\ &= \underset{f_{\text{imp}}}{\arg\max}(UAR(Y,h(\hat{X}^{\text{nomiss}}))) \\ &= \underset{f_{\text{imp}}}{\arg\max}(UAR(Y,h(f_{\text{imp}}(X))) \end{aligned}$$

with:

- UAR is unweighted average recall
- X is the training dataset containing NULL values and Y is the associated ASD diagnosis
- X^{nomiss} is the theoretical version of X containing "correct" missing values
- $\hat{X}^{\text{nomiss}}$ is our estimation of X^{nomiss} using feature imputation method f_{imp}
- h is the ASD classifier, either LR9 or ADTree7, with a MinMaxScaler

We then test on a record Z containing missing values by establishing an ASD prediction using both f^*_{imp} and h:

$$\text{ASD prediction for } Z = h(f^*_{\text{imp}}(Z))$$

General feature replacement methods

Let us consider Z a new test record for which we wish to predict ASD class and that has a missing value in feature j. Our estimation of $\hat{z}_j^{\text{nomiss}}$ will be z_{j*} if feature j^* is the closest feature to j as per score s in the training set X. Using mutual information (MI), for example, the replaced feature would be:

$$j^* = \underset{l \in [\text{ all } n^* \text{ features available }]}{\arg\max} (MI(X_j, X_l))$$

Dynamic feature replacement

Let us consider Z a new test record for which we wish to predict ASD class and that has a missing value in feature j. Our estimation of $\hat{z}_j^{\text{nomiss}}$ will be z_{j*} if feature j^* is the closest feature to j as per score s in the subset of the training set $\tilde{X}$. Using mutual information (MI), for example, the predicted feature would be:

$$j^* = \underset{l \in [\text{ all } n^* \text{ features available }]}{\arg\max} (MI(\tilde{X}_j, \tilde{X}_l))$$

$\tilde{X}$ is defined as all records in X with a similar rating of Z, i.e. for all features l in Z, $x_{i,l}$ is in a -1/+1 range of z_l based on the ADOS or ADI-R questions' ordinal scale.

$$\tilde{X} = \{\text{all records } i \text{ in } X \text{ such that } \forall l, x_{i,l} - 1 \le z_l \le x_{i,l} + 1\}$$

As defined above, $\tilde{X}$'s size varies the number of ratings in the training set similar to Z.

References

1. Stekhoven, D. J. & Bühlmann, P. Missforest—non-parametric missing value imputation for mixed-type data. *Bioinformatics* **28**, 112–118 (2012).

Figures and Tables

Diagnosis / Gender	Female	Male
ASD	594	2,883
NT	201	419

(a) ADOS Module 2 - LR9 training dataset

Diagnosis / Gender	Female	Male
ASD	1,897	9,409
NT	191	241

(b) ADI-R 2003 - ADTree7 training dataset

Table 1. Gender and Diagnosis in training datasets

Age / Diagnosis	<1	1-3	4-6	7-10	11-15	16-18	>18	NULL
ASD	172	452	1,439	698	229	31	33	610
NT	124	331	184	29	8	1	1	1

(a) ADOS Module 2 - LR9 training dataset

Age / Diagnosis	<1	1-3	4-6	7-10	11-15	16-18	>18	NULL
ASD	13	1,010	2,530	2,567	1,659	403	343	2,896
NT	0	70	146	119	81	10	9	1

(b) ADI-R 2003 - ADTree7 training dataset

Table 2. Age Group and Diagnosis in training datasets (age in years)

Diagnosis / Gender	Female	Male
ASD	26	44
NT	32	38

(a) Gender and Diagnosis

Diagnosis / Age	<1	1-3	4-6	7-10	11-15	16-18	>18	NULL
ASD	0	32	34	4	0	0	0	0
NT	0	43	26	1	0	0	0	0

(b) Age Group and Diagnosis (age in years)

Table 3. YouTube testing dataset description

**Rated feature**	**Used in LR9**	**Used in ADTree7**
Echolalia		
Expressive Language	B10 Amount of reciprocal social communication	35 - conver5 Reciprocal conversation of simple language: answer most abnormal between 4 and 5
Speech Patterns		
Communicative Engagement		
Aggression		
Entertains Self		
Understands Language		29 - compsl5 Comprehension of simple language: answer most abnormal between 4 and 5
Eye Contact	B01 Unusual eye contact	50 - gaze5 Direct gaze: answer most abnormal between 4 and 5
Responsiveness		

Developmental Delay		86 - ageabn<br>Age when abnormality first evident
Comforts Others		
Social Participation		64 - grplay5<br>Group play with peers:<br>answer most abnormal between 4 and 5
Sensory Aversion		
Imitates Actions		
Emotion Expression		
Sensory Seeking		
Pretend Play		48 - play5<br>Imaginative play:<br>answer most abnormal between 4 and 5
Shakes Head YesNo		
Responsive Social Smile		
Calls Attention to Objects		
Joint Attention Pointing	B06<br>Spontaneous initiation<br>of joint attention	
Appropriate Play		
Creativity		
Stereotyped Speech	A05<br>Stereotyped-idiosyncratic<br>use of words or phases	
Spontaneous Gestures	A08<br>Descriptive conventional instrumental<br>or informative gestures	
Indicates Pleasure to Others	B03<br>Shared enjoyment in interaction	49 - peerpl5<br>Imaginative play with peers:<br>answer most abnormal between 4 and 5
Social Overtures	B08<br>Quality of social overtures	
Complex Mannerisms	D02<br>Hand and finger and<br>other complex mannerisms	
Stereotyped Interests Actions	D04<br>Unusually repetitive interests<br>or stereotyped behaviors	

Table 4. 30 features scored by video raters and, if they are features of the LR9 or ADTree7 algorithms, their mapped ADOS and ADI-R features

LR9 Feature<br>*Rated feature used*	ADOS Module 2 Replacement feature<br>*Rated Replacement feature used*
B10 - Amount of reciprocal social communication<br>*Rated feature used: Expressive Language*	A02 - Amount of social overtures / maintenance of attention<br>*Rated feature used: Social Overtures*
D02 - Hand and finger and other complex mannerisms<br>*Rated feature used: Complex mannerisms*	D01 - Unusual sensory interest in play material / person.<br>*Rated feature used: Sensory Seeking*
A08 - Descriptive conventional instrumental or informative gestures<br>*Rated feature used: Spontaneous gestures*	B10 - Amount of reciprocal social communication<br>*Rated feature used:*
B08 - Quality of social overtures<br>*Rated feature used: Social overtures*	A02 - Amount of social overtures / maintenance of attention<br>*Rated feature used: Social Overtures*
D04 - Unusually repetitive interests or stereotyped behaviors<br>*Rated feature used: Stereotyped Interests Actions*	D01 - Unusual sensory interest in play material / person.<br>*Rated feature used: Sensory Seeking*
B03 - Shared enjoyment in interaction<br>*Rated feature used: Indicates Pleasure to Others*	A02 - Amount of social overtures / maintenance of attention<br>*Rated feature used: Social Overtures*
B06 - Spontaneous initiation of joint attention<br>*Rated feature used: Joint Attention Pointing*	A07 - Pointing<br>*Rated feature used: Calls Attention to Objects*
A05 - Stereotyped/idiosyncratic use of words or phases<br>*Rated feature used: Stereotyped Speech*	B09 - Quality of social response<br>*Rated feature used: Responsiveness*
B01 - Unusual eye contact<br>*Rated feature used: Eye contact*	B08 - Quality social overtures<br>*Rated feature used: Social Overtures*

Table 5. LR9 Features and replacements - correlation based selection

ADTree7 Feature<br>*Rated feature used*	ADI-R 2003 Replacement feature<br>*Rated Replacement feature used*
86 - Age when abnormality first evident<br>*Rated feature used: Developmental Delay*	69.2 - Ever repetitive use of objects or interests in parts of objects<br>*Rated feature used: Stereotyped Interests Actions*
29 - Comprehension of simple language: answer most abnormal between 4 and 5<br>*Rated feature used: Understands Language*	41.2 - At 5 current communicative speech<br>*Rated feature used: Expressive Language*
48 - Imaginative play: answer most abnormal between 4 and 5<br>*Rated feature used: Pretend Play*	49 - Imaginative play with peers: answer most abnormal between 4 and 5<br>*Rated feature used: Indicates Pleasure to Others*
49 - Imaginative play with peers: answer most abnormal between 4 and 5<br>*Rated feature used: Indicates Pleasure to Others*	48 - Imaginative play: answer most abnormal between 4 and 5<br>*Rated feature used: Pretend Play*
64 - Group play with peers: answer most abnormal between 4 and 5<br>*Rated feature used: Social Participation*	63.2 - At 4-5 response to approaches of other children<br>*Rated feature used: Comforts Others*
35 - Reciprocal conversation of simple language: answer most abnormal between 4 and 5<br>*Rated feature used: Expressive Language*	34.2 - Ever social verbalization / chat<br>*Rated feature used: Understands Language*
50 - Direct gaze: answer most abnormal between 4 and 5<br>*Rated feature used: Eye contact*	56.2 - At 4-5 quality of social overtures<br>*Rated feature used: Social Overtures*

Table 6. ADTree7 Features and replacements - correlation based selection

**LR9 Feature**<br>*Rated feature used*	**ADOS M2 Replacement feature**<br>*Rated Replacement feature used*
B10 - Amount of reciprocal social communication<br>*Rated feature used: Expressive Language*	B09 - Quality of social response<br>*Rated feature used: Social Participation*
D02 - Hand and finger and other complex mannerisms<br>*Rated feature used: Complex mannerisms*	D01 - Unusual sensory interest in play material/person<br>*Rated feature used: Sensory Seeking*
A08 - Descriptive conventional instrumental<br>or informative gestures<br>*Rated feature used: Spontaneous gestures*	B10 - Amount reciprocal social communication<br>*Rated feature used: Expressive Language*
B08 - Quality of social overtures<br>*Rated feature used: Social overtures*	B09 - Quality of social response.<br>*Rated feature used: Social Participation*
D04 - Unusually repetitive interests<br>or stereotyped behaviors<br>*Rated feature used: Stereotyped Interests Actions*	D01 - Unusual sensory interest in play material/person.<br>*Rated feature used: Sensory Seeking*
B03 - Shared enjoyment in interaction<br>*Rated feature used: Indicates Pleasure to Others*	A02 - Amount of social overtures/maintenance of attention.<br>*Rated feature used: Responsiveness*
B06 - Spontaneous initiation of joint attention<br>*Rated feature used: Joint Attention Pointing*	A02 - Amount of social overtures/maintenance of attention.<br>*Rated feature used: Responsiveness*
A05 - Stereotyped/idiosyncratic use of words or phases<br>*Rated feature used: Stereotyped Speech*	B09 - Quality of social response.<br>*Rated feature used: Social Participation*
B01 - Unusual eye contact<br>*Rated feature used: Eye contact*	A06 - Conversation.<br>*Rated feature used: Understands Language*

Table 7. LR9 Features and replacements - nearest neighbor selection

**ADTree7 Feature**<br>*Rated feature used*	**ADI-R 2003 Replacement feature**<br>*Rated Replacement feature used*
86 - Age when abnormality first evident<br>*Rated feature used: Developmental Delay*	35 - Reciprocal conversation of simple language:<br>answer most abnormal between 4 and 5<br>*Rated feature used: Expressive Language*
29 - Comprehension of simple language:<br>answer most abnormal between 4 and 5<br>*Rated feature used: Understands Language*	54.2 - At 4-5 seeking to share his/her enjoyment with others<br>*Rated feature used: Shares Excitement*
48 - Imaginative play:<br>answer most abnormal between 4 and 5<br>*Rated feature used: Pretend Play*	49 - Imaginative play with peers:<br>answer most abnormal between 4 and 5<br>*Rated feature used: Indicates Pleasure to Others*
49 - Imaginative play with peers:<br>answer most abnormal between 4 and 5<br>*Rated feature used: Indicates Pleasure to Others*	48 - Imaginative play:<br>answer most abnormal between 4 and 5<br>*Rated feature used: Pretend Play*
64 - Group play with peers:<br>answer most abnormal between 4 and 5<br>*Rated feature used: Social Participation*	49 - Imaginative play with peers:<br>answer most abnormal between 4 and 5<br>*Rated feature used: Indicates Pleasure to Others*
35 - Reciprocal conversation of simple language:<br>answer most abnormal between 4 and 5<br>*Rated feature used: Expressive Language*	34.2 - Ever social verbalization/chat<br>*Rated feature used: Understands Language*
50 - Direct gaze:<br>answer most abnormal between 4 and 5<br>*Rated feature used: Eye contact*	63.2 - At 4-5 response to approaches of other children<br>*Rated feature used: Social Participation*

Table 8. ADTree7 Features and replacements - nearest neighbor selection

**LR9 Feature**<br>*Rated feature used*	**ADOS M2 Replacement feature**<br>*Rated Replacement feature used*
B10 - Amount of reciprocal social communication<br>*Rated feature used: Expressive Language*	A02 - Amount of social overtures/maintenance of attention<br>*Rated feature used: Social Overtures*
D02 - Hand and finger and other complex mannerisms<br>*Rated feature used: Complex mannerisms*	D01 - Unusual sensory interest in play material/person<br>*Rated feature used: Sensory Seeking*
A08 - Descriptive conventional instrumental or informative gestures<br>*Rated feature used: Spontaneous gestures*	A06 - Conversation<br>*Rated feature used: Communicative Engagement*
B08 - Quality of social overtures<br>*Rated feature used: Social overtures*	A02 - Amount of social overtures/maintenance of attention<br>*Rated feature used: Social Overtures*
D04 - Unusually repetitive interests or stereotyped behaviors<br>*Rated feature used: Stereotyped Interests Actions*	D01 - Unusual sensory interest in play material/person<br>*Rated feature used: Sensory Seeking*
B03 - Shared enjoyment in interaction<br>*Rated feature used: Indicates Pleasure to Others*	A02 - Amount of social overtures/maintenance of attention<br>*Rated feature used: Social Overtures*
B06 - Spontaneous initiation of joint attention<br>*Rated feature used: Joint Attention Pointing*	A07 - Pointing<br>*Rated feature used: Calls Attention to Objects*
A05 - Stereotyped/idiosyncratic use of words or phases<br>*Rated feature used: Stereotyped Speech*	B09 - Quality of social response<br>*Rated feature used: Responsiveness*
B01 - Unusual eye contact<br>*Rated feature used: Eye contact*	B09 - Quality of social response<br>*Rated feature used: Responsiveness*

Table 9. LR9 Features and replacements - mutual information selection

**ADTree7 Feature**<br>*Rated feature used*	**ADI-R 2003 Replacement feature**<br>*Rated Replacement feature used*
86 - Age when abnormality first evident<br>*Rated feature used: Developmental Delay*	04a - Coded: Onset as perceived with hindsight<br>*Rated feature used: Developmental Delay*
29 - Comprehension of simple language: answer most abnormal between 4 and 5<br>*Rated feature used: Understands Language*	41.2 - At 5 current communicative speech<br>*Rated feature used: Communicative Engagement*
48 - Imaginative play: answer most abnormal between 4 and 5<br>*Rated feature used: Pretend Play*	47.2 - At 4-5 spontaneous imitation of actions<br>*Rated feature used: Imitates Actions*
49 - Imaginative play with peers: answer most abnormal between 4 and 5<br>*Rated feature used: Indicates Pleasure to Others*	56.2 - At 4-5 response to approaches of other children<br>*Rated feature used: Social Overtures*
64 - Group play with peers: answer most abnormal between 4 and 5<br>*Rated feature used: Social Participation*	63.2 - At 4-5 response to approaches of other children<br>*Rated feature used: Social Participation*
35 - Reciprocal conversation of simple language: answer most abnormal between 4 and 5<br>*Rated feature used: Expressive Language*	34.2 - Ever social verbalization/chat<br>*Rated feature used: Understands Language*
50 - Direct gaze: answer most abnormal between 4 and 5<br>*Rated feature used: Eye contact*	56.2 - At 4-5 quality of social overtures<br>*Rated feature used: Social Overtures*

Table 10. ADTree7 Features and replacements - mutual information selection

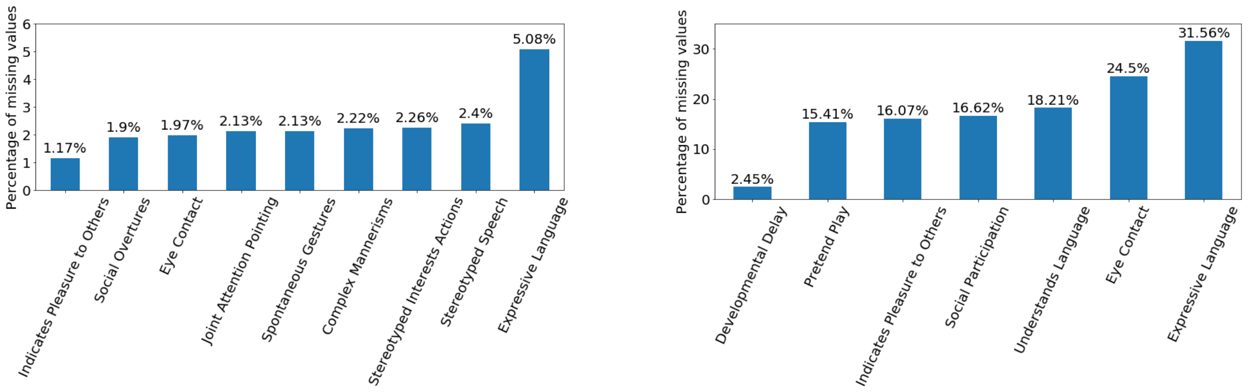

(a) LR9 - ADOS M2 training dataset

(b) ADTree7 - ADI-R training dataset

Figure 1. Percentage of missing values per model feature in training datasets

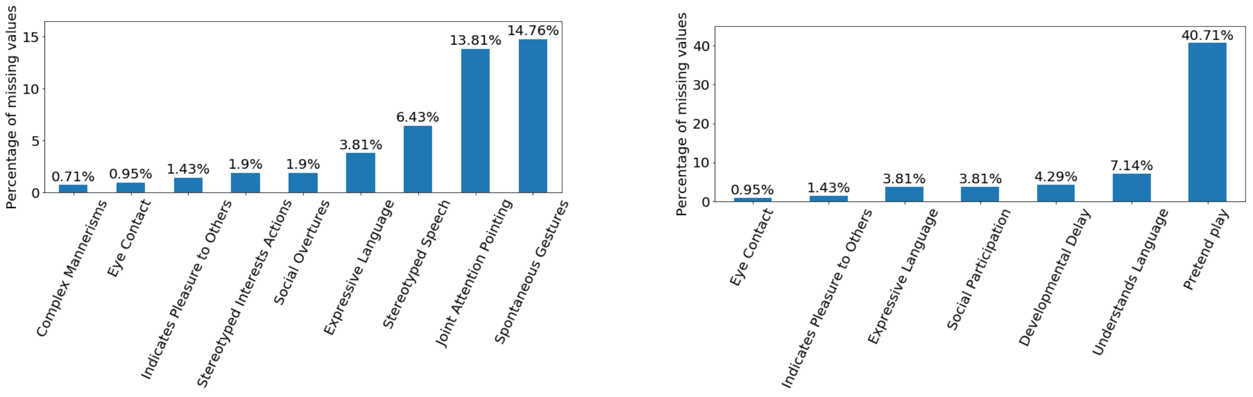

(a) LR9 - YouTube ratings testing dataset

(b) ADTree7 - YouTube ratings testing dataset

Figure 2. Percentage of missing values per model feature in testing datasets
